# Exploring Black and South Asian women’s experiences of help-seeking and engagement in perinatal mental health services in the UK

**DOI:** 10.3389/fpsyt.2023.1119998

**Published:** 2023-04-03

**Authors:** Maev Conneely, Katy C. Packer, Sarah Bicknell, Jelena Janković, Harpreet Kaur Sihre, Rosemarie McCabe, Alex Copello, Kiren Bains, Stefan Priebe, Amy Spruce, Nikolina Jovanović

**Affiliations:** ^1^Unit for Social and Community Psychiatry, WHO Collaborating Centre, Wolfson Institute of Population Health, Queen Mary University of London, London, United Kingdom; ^2^East London NHS Foundation Trust, London, United Kingdom; ^3^Camden and Islington NHS Foundation Trust, London, United Kingdom; ^4^North East London NHS Foundation Trust, London, United Kingdom; ^5^Birmingham and Solihull Mental Health Foundation Trust, Birmingham, United Kingdom; ^6^School of Psychology, University of Birmingham, Birmingham, United Kingdom; ^7^Department of Applied Health Research, University College London, London, United Kingdom; ^8^School of Health and Psychological Sciences, City, University of London, London, United Kingdom; ^9^Action on Postpartum Psychosis, London, United Kingdom

**Keywords:** health inequality, framework analysis (FWA), qualitative study, ethnic minority, perinatal mental health, marginalized and vulnerable groups, minoritisation

## Abstract

**Background and aims:**

In the United Kingdom (UK), Black and South Asian women are less likely than White British women to access support from perinatal mental health services, despite experiencing similar, or higher, levels of distress. This inequality needs to be understood and remedied. The aim of this study was to answer two questions: how do Black and South Asian women experience (1) access to perinatal mental health services and (2) care received from perinatal mental health services?

**Method:**

Semi-structured interviews were conducted with Black and South Asian women (*n* = 37), including four women who were interviewed with an interpreter. Interviews were recorded and transcribed line-by-line. Data were analyzed using framework analysis, by an ethnically diverse multidisciplinary team of clinicians, researchers and people with lived experience of perinatal mental illness.

**Results:**

Participants described a complex interplay of factors that impacted on seeking, and receiving help, and benefiting from services. Four themes emerged that captured the highly varied experiences of individuals: (1) Self-identity, social expectations and different attributions of distress deter help-seeking; (2) Hidden and disorganized services impede getting support; (3) The role of curiosity, kindness and flexibility in making women feel heard, accepted and supported by clinicians; (4) A shared cultural background may support or hinder trust and rapport.

**Conclusion:**

Women described a wide range of experiences and a complex interplay of factors impacting access to, and experience of, services. Women described services as giving them strength and also leaving them disappointed and confused about where to get help. The main barriers to access were attributions related to mental distress, stigma, mistrust and lack of visibility of services, and organizational gaps in the referral process. These findings describe that many women feel heard, and supported by services, reporting that services provide a high quality of care that was inclusive of diverse experiences and understandings of mental health problems. Transparency around what PMHS are, and what support is available would improve the accessibility of PMHS.

## 1. Introduction

Since 2015 national reports in the UK have continuously shown an increase in maternal death rate (1). A report on maternal deaths from 2020 attributed this rise to an increase in suicide-related deaths and COVID-19-related deaths (2). Women from Black ethnic backgrounds are three times more likely to die in the perinatal period than White women, and women from Asian ethnic backgrounds are twice as likely to die compared to White women, with the leading causes of death identified as obstetric hemorrhage, pregnancy related sepsis and suicide. Assessors judged that only 17% of the women who died had good care, and that improvements in care could have made a difference to the outcome in 37% of the women who died (2). National cohort studies show that mothers from ethnic minority groups are less likely to receive treatment for anxiety and depression in the perinatal period than White British mothers, despite experiencing similar levels of psychological distress (3). A recent study, which was conducted as part of the larger programme of research this study is a part of, found that specifically South Asian women, Black women, and White other women accessed community mental health perinatal mental health services (PMHS) less than White British women and had higher rates of involuntary admission (4). This health inequality is not easily explained. There is a clear, urgent need for continued and focused action to address these disparities and provide better support to women in the perinatal period, in particular Black and South Asian women who are least likely to access support (5, 6).

Ethnicity is multifaceted and complex, and grouping in a limited set of categories is flawed and reductive. However, not using categories risks masking health inequalities and inequities and would prevent remedying the disparities currently observed. In this study, “South Asian” is used, as a broad ethnic category to refer to women whose cultural or familial backgrounds originate from the subcontinent of India, Pakistan, Bangladesh and Sri Lanka, following Marshall and Yazdani’s (7) definition. The categorization for this study was taken from the British census, for ease of comparison across studies, despite the limitations of using a pre-determined category-based approach. According to the 2021 census, the most common high-level minority ethnic group in England and Wales was “Asian, Asian British or Asian Welsh” accounting for 9.3% (5.5 million) of the overall population (8). Research identifying South Asian women as having higher rates of distress, and lower access to services is not new (9–11), with data coming from large scale community studies pointing to a vulnerability to depression in particular subgroups of South Asian women. A seminal study showed that rates of attempted suicide for South Asian women were 1.5 times higher than those for White women (12). Reviews of the mental health of South Asian women describe specificities of different subgroups across the country, generally indicating increased vulnerability that is not matched by the level of support provided by services (13–15).

Less research has examined Black women’s access to mental health services in the UK (16). According to the census in 2021, 2.5% (1.5 million) of the population in England and Wales identify in the high-level category “Black” (8). The research that exists shows evidence of Black women being excluded in terms of access to support (17), and that Black women are left out of healthcare research (18–20). There is even less research examining Black women’s experiences of perinatal mental healthcare (16, 21).

The lower rates of access to community help in Black and South Asian mothers is unlikely to represent not needing help, as evidenced in the higher rates of emergency and involuntary admissions (4). Furthermore, accessing community support sooner could reduce the number of highly distressing emergency and involuntary admissions, which have been described as traumatizing in themselves (22). There is some evidence for delayed help-seeking in ethnic minority groups in the UK, and other Westernized English-speaking countries such as Canada, however, the evidence is mixed, with certain studies showing similar help-seeking behavior across ethnic groups (23). Differences in help-seeking cannot wholly explain differences in engagement with care in women with moderate to severe perinatal distress. A systematic review examining whether the UK guidance on detection and management of perinatal mental health problems is equitable, found that the most consistent health disparity was between ethnic minority women and the White British majority (24). Taken together, these findings suggest differences in help-seeking cannot wholly explain differences in engagement with care in women with perinatal distress. Calls for change and improved understanding have come from public health research, clinical research and government (25–27).

Exploring women’s experiences will allow for a much-needed understanding of the current situation to be developed: how do women experience services and potential barriers to coming into contact with services? What is not working well within the way services are run? What is working well? What can be done to improve services provided? The present study aimed to answer the following research questions: how do Black and South Asian women experience (1) access to perinatal mental health services and (2) care received from perinatal mental health services?

## 2. Materials and methods

This study was approved by the Health Research Authority *via* London–Queen Square Research Ethics Committee (19/LO/1830). All participants provided informed consent for the publication of anonymized quotations included in this article. Participants were recruited through East London NHS Foundation Trust (ELFT), North East London National Health Service (NHS) Foundation Trust (NELFT), Devon Partnership NHS Trust, and Birmingham and Solihull Mental Health Foundation Trust (BSMHFT). The study is reported according to the COREQ guidelines (28) (see Supplementary material 1), and was designed by JJ, NJ, and SP with input from a Lived Experience Advisory Panel (LEAP) of women who had experienced perinatal mental illness assembled for the study, which included women from diverse backgrounds, including South Asian and Black women (the topic guide can be found in Supplementary material 2). The study is registered on the Open Science Framework: https://osf.io/s94bp/.

### 2.1. Procedure and inclusion

Members of multi-disciplinary clinical care teams identified potential participants, approached them and obtained consent to provide the research team with their contact details. Eligible participants met the following criteria: (1) having the ability to provide informed consent, (2) aged 16 or above, (3) identify their ethnicity as South Asian (i.e., Indian/Bangladeshi/Pakistani) and/or Black (i.e., Black African/Black Caribbean/Black Other); (4) have experiences of perinatal mental health problems (pregnancy and first postnatal year) of moderate-severe intensity in the last 2 years (the severity was based on health professional’s judgment); (5) be actively involved in the care of their baby and accessed support from PMHS. For the purpose of this study “access” is defined as having attended at least one appointment with PMHS. There were no exclusion criteria.

### 2.2. Sampling

Non-probabilistic, purposive sampling was used to maximize variation within the key sample criteria of ethnicity, migration experience (e.g., migrants, people whose parents were not born in the UK, and people whose parents were all born in the UK); ability to speak English. Grouping women by high-order ethnic groups using the British census categories was done for ease of comparison across studies. Efforts were made to include people who did not speak English fluently, as they are often left voiceless in research studies.

Thematic saturation, the point at which no novel insights arise from the data, is a contentious topic in qualitative analysis, with views diverging as to the point at which it tends to occur, and whether that point exists (29, 30). Researchers have found that from 12 participants within a homogenous group, about a specific topic, data saturation begins to occur. For this study, as the group was not homogenous, to be conservative, an *a priori* decision was made to recruit sample size of between 20–30 participants (31, 32). Throughout the coding of the data, researchers noted the rate of new ideas arising, with the option to increase the number of participants recruited if deemed necessary. A subset of participants were recruited who did not speak English fluently, and who were interviewed with the help of professional interpreters. There were no drop-outs, and no record was kept of reasons people did not want to participate.

### 2.3. Data collection

Semi-structured interviews were conducted over the phone, or video call, between January 2020 and August 2021 by four researchers (SB, KCP, HKS, and AS, see full details of interviewers in Supplementary material 3), one of whom had lived experience of perinatal mental illness. Mostly interviews were one-to-one, however, occasionally women were interviewed with their children and or partners present. Women were describing experiences of care received both during and before the pandemic, and most of the interviews took place remotely during or between national lockdowns in the UK. The topic guides (Supplementary material 2) explored how they received treatment (remote or in person) and when they received treatment whether there was a national lockdown or not, whether they felt that the format of care and the method of interviewing had an impact on their experience of care. Details of the interviewers can be found in the reflexivity section and in Supplementary material 3. Women were given a £30 shopping voucher to reimburse the time taken for participation.

### 2.4. Data analysis

Interviews were audio-recorded and transcribed verbatim by an external transcription agency. Data were analyzed using framework analysis, an approach to qualitative data analysis that was developed for the explicit purpose of analyzing qualitative data in applied policy research. Framework analysis was chosen as it is particularly well-suited to the context of multidisciplinary health research (33). It is an inherently comparative form of thematic analysis which employs a flexible, organized structure of inductive and deductive coding, obtained in five distinct steps (34). Three members of the research team (KCP, HKS, and AS) first read a selection of transcripts several times to become familiar with the data (familiarization). An initial framework was developed using a combination of *a priori* concerns, such as those included in the topic guide, and emergent themes from the data (identifying a framework). The framework was systematically applied to the transcripts and the framework was adapted as new codes emerged (indexing). Data were then summarized in a matrix, where each participant was allocated a row and each subtheme was allocated a column (charting). HKS was involved in the stages of the analysis up until mapping and interpretation. Three researchers (KCP, SB, and MC) then met regularly to review the charted material and develop emergent themes (mapping and interpretation). The themes were regularly reviewed in discussion with the wider analysis team (JJ, NJ, AC, and RM).

The analysis was conducted from a pragmatic worldview, as this is aligned with the needs of the project (i.e., understanding accessibility and acceptability), and with patient-oriented care (35). Pragmatic worldviews are often adopted in healthcare research which aims to produce knowledge in a format most useful for influencing practice, policy and to inform recommendations for clinical practice. Additional questions about ethnicity and culture and the role it played guided the analysis beyond the research questions [i.e., how do Black and South Asian women experience (1) access to perinatal mental health services and (2) care received from perinatal mental health services?]. These were: How do women perceive their ethnicity and culture impacting their experiences of services and how could the acceptability and accessibility of services be improved in relation to culture and ethnicity?

### 2.5. Reflexivity

The characteristics of the researchers conducting the analysis can be found in Supplementary material 3. Throughout the analysis reflective and reflexive practices were employed to identify leanings, biases and other factors that may have had a bearing on how the data were interpreted (36). No relationship was established with the participants before the study commenced and it was made clear to participants that the researchers were not part of the clinical team and what they said in interviews would not be communicated to the clinical team.

HKS, SB and KCP were involved in data interpretation. AS, hired by the charity Action on Postpartum Psychosis, conducted interviews and was involved in the first two stages of analysis. MC re-listened to each of the interviews in turn to identify key features and find contradictory evidence to the framework as a robustness test (37).

### 2.6. Language

The langued used to describe the social constructs of group belongings, ethnicity and race is highly emotive and powerful, and has different connotations at different times and across different contexts. In this paper race and ethnicity are understood as “dynamic, shaped by geographic, cultural, and sociopolitical forces,” as defined in recent guidance (38). Throughout the paper we use this guidance and the NHS Race Networks’ guidance in describing ethnic groups. The language for women’s ethnic groups in this study was taken from women’s self-categorization using the UK census categories. There are problems with this approach that are inherent to attempting to reducing complex concepts of identity and belonging to simple categories (39, 40). However, using these categories makes comparison across studies easier and avoids the risk of disregarding or concealing health disparities, injustices, and inequities. Using standardized categories makes it easier for different pieces of work to be brought together to target and change these health disparities (41, 42). Throughout the results women’s ethnic group is reported alongside each quote, as it may provide important context to understand the lived experience of the woman, and although it does not provide much by way of explaining that woman’s relationship with her ethnicity and culture, it does provide some information about the racialized minority identity she is likely have gone through PMHS (and life) with.

## 3. Results

### 3.1. Participant characteristics

Thirty-seven women were interviewed. A breakdown of their demographic characteristics can be found in Table 1. Of the women interviewed, 46% were born in the UK. Four women were interviewed with the help of an interpreter. The languages of women who could not speak English fluently were Bengali (*n* = 2) and Urdu (*n* = 2). Interviews lasted between 23 and 65 min. Diagnoses were self-reported and fell in the following categories: depression/postnatal depression (*n* = 17), anxiety disorders (*n* = 16), Post-Traumatic Stress Disorder (*n* = 9), personality disorders (*n* = 5), bipolar disorder (*n* = 4), and psychosis (*n* = 2).^1^ Most participants were recruited in Birmingham and East London, and two were recruited in Devon.

**TABLE 1:** Demographic characteristics.

	*n*	% of sample
**Age range**		
20–29	11	30%
30–39	25	67%
40–49	1	3%
**Countries of birth**		
Austria	1	3%
Bangladesh	4	10%
Bermuda	1	3%
Eritrea	1	3%
Germany	2	5%
Ghana	1	3%
India	3	8%
Jamaica	1	3%
Nigeria	3	8%
Pakistan	2	5%
UK	18	48%
**Ethnicity**		
Black African	9	24%
Black Caribbean	6	16%
Black Caribbean and Black African	1	3%
Asian Bangladeshi	8	21%
Asian Pakistani	6	16%
Asian Indian	3	8%
White and Black Caribbean	3	8%
White and Asian	1	3%
**Religion**		
Muslim	17	45%
Christian	13	33%
No religion	5	13%
Rastafarian	1	3%
Sikh	1	3%
**Partner?**		
Married or cohabiting	21	56%
Single	9	24%
Partner but not cohabiting	4	11%
Separated or divorced	1	3%
Not known	2	5%

### 3.2. Overview

**FIGURE 1:**
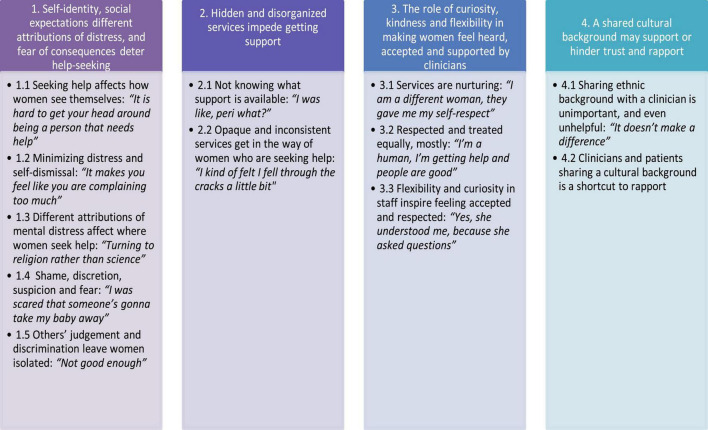
Themes and subthemes.

Themes reflect a broad range of experiences that are in certain respects polarized (Figure 1; Supplementary material 4). The apparent contradictions across themes speak to this diversity in views and the complex range of experiences. Four themes describe how women accessed and experienced care. Threaded through all of the themes and subthemes was the interactive nature of the factors impacting women’s experiences of services, both accessing them and their experiences once they were in services. Most women did not know about services existing. Only five out of the 37 had heard of PMHS before accessing support. Women mostly received care in person, despite the context of the lockdowns and most women felt appreciative of someone coming to their homes. The quotes in the following section are accompanied with the participant number and ethnicity of the speaker to provide context.

## 4. Theme 1: Self-identity, social expectations, and different attributions of distress deter help-seeking

Mis-trust of services, stigma and discrimination coalesced to deter help-seeking, leaving women suffering in silence. A complex interplay between personal, social, and cultural factors were described as having an important impact on women’s help-seeking behavior. These factors and pressures came from inside (i.e., women’s thoughts about their self-identity), and outside, in intertwining layers that were difficult to distinguish. Some of these layers included friends’ and family members’ views on help-seeking, women’s own expectations that they should not need help from services, and the stigma attached to both having mental health problems and having any involvement with mental health, or social, services.

### 4.1. Seeking help affects how women see themselves: “*It is hard to get your head around being a person that needs help”*

Women described how they saw themselves and cultural expectations as barriers to asking for help. Participant 10 felt a pressure to be strong, saying: “*I’m Black, (laughs) I’m Black and I’m Caribbean. They don’t expect you to be weak.”* She described asking for support with her mental health as in conflict with what fell under the remit of “being strong.” Women described an internal barrier in admitting to themselves that they needed help, and that it was a journey to get to the stage where they feel able to ask for help: “*It* (seeking help from services) *was the right thing to do, but it was a hard thing to do. It’s hard to* (hesitation) *it is hard to get your head around being a person that needs help*” (Participant_3, Asian Pakistani).

Seeking help was described as particularly difficult as, for many it was described as coming with a need to accept that they would be judged by others in their families who associated mental health problems with shame. Women described a journey to come to a place where they did not care about what others thought, and rather prioritized doing what was best for their health and for their children.

*“I like to believe that I’m very open-minded. I don’t believe in being tough or being strong, I believe that we are all strong and tough but sometimes we need a little soft hand. And so, I am not afraid to ask for help when I need it. So, when I was getting the help I needed I thought yes, this is what I needed, and I wasn’t ashamed of it, you know, because of this misconception of ‘be strong, be tough’.”* (Participant_23, Black African).

### 4.2. Minimizing distress and self-dismissal: “*It makes you feel like you are complaining too much”*

Many women felt their expectations of themselves were sometimes reinforced by things their family said which, even if it was not their intention, often resulted in their distress being minimized or dismissed. They felt that they should not be struggling, and should toughen up and shrug off feelings of distress, and not complain.

*“In my culture it’s usually, they don’t. They wouldn’t, I dunno I feel like sometimes they wouldn’t take it that serious mental health*… *they think I’m just being down”* (Participant_14, Asian Pakistani).

*“In the culture they don’t really believe in mental health and stuff like that and if you were to tell you were having problems and stuff they’d be like ‘it’s fine,’ ‘it’s nothing’ and stuff like that. I find that a bit harsh to be honest, cos someone not understanding you, I just feel I get worse when people don’t understand me, I get even more depressed and I tend to like push myself away from people even more because no one is understanding me”* (Participant_30, Asian Bangladeshi).

Women described their family’s reactions to sharing feelings as making them feel like they should not have shared, or were making a big deal out of nothing: *“It makes you feel like you are complaining too much, like you should not be feeling the way you are feeling”* (Participant_28, Black African).

Concerns that their family members and friends had voiced about them receiving support from mental health services also prevented seeking help. The most common concern that family members and friends had were that mental health services, including PMHS, would remove their children. Several participants described their close family as atypical, and non-representative of the wider communities’ views:

*“That was one of the first things actually my family said, they were like, ‘oh you know they’ll take your baby away’* (…) *and if it wasn’t for my husband insisting I get help I don’t think I would have done it. He’s very different, he’s British born and his family is very backward but he’s very different, he’s very, I don’t know, he’s kind of like White in an Asian person’s body. If it wasn’t for him, I wouldn’t have done it because my family would have discouraged me for sure”* (Participant_6, Asian Indian).

Women felt that their families had internalized the expectation that suffering is normal, and thus seeking help to reduce suffering was an alien concept. Women saw this expectation as having an important impact on their not seeking help from services. The importance of changing these expectations to be able to seek and accept help was highlighted by Participant 9.

“I think I’ve done a lot of reconditioning from my Jamaican culture. I love my culture very dearly, but I think that Jamaicans especially forget that our country was made from slaves. So there’s a lot of things that we have accepted as normal that ain’t f^**^king normal” (Participant_9, Black Caribbean).

Changing the expectation that pain and suffering is normal, which was contextualized in the history of discrimination and suffering experienced by older generations, was described as hard work that women had intentionally decided to do. The process of changing expectations, to be able to ask for and accept help, and trust services, was seen as important.

### 4.3. Different attributions of mental distress affect where women seek help: “*Turning to religion rather than science”*

Opposition between the medical ways services understood distress, and spiritual ways of understanding distress, was described as a barrier to help-seeking and as personally difficult to reconcile. Understanding distress in a non-medical way meant the solution was to seek support from non-medical avenues. Women described how they were often advised seek help from their communities, especially religious leaders, and were told to *“go to the Mullah”* (Participant_31, Asian Bangladeshi), or *“go to your local mosque or your local Gurdwara, you know, go to your friends and family*” (Participant_3, Asian Pakistani). One woman felt it is unlikely that she would have sought help if her midwife had not made the referral to PMHS, as described below:

*“I don’t know if I would have gotten any help for things that were stressing me out if she [midwife] hadn’t offered to make the referral* (…) *Because I know that with women of color, especially in my family, mental health isn’t something that you get help for, it’s more if you’re suffering from anything then we turn to religion rather than science”* (Participant_11, Black African).

Women spoke about turning to religion for support over medical professionals, and the non-medical interpretations of emotional distress as a source of difficulty and a barrier to getting help. Mental health problems were interpreted by family members, especially older people, in ways that were non-medical, and not in alignment with the language used by services: “*They only know what their parents told them which is you know, ‘oh she could be possessed’ or something like that”* (Participant_2, Asian Bangladeshi).

*“Even my mum that loves me dearly, I watched her struggle to know how to say that I was ill or that I had an illness. It was straightaway something to do with the devil”* (Participant_10, Black Caribbean).

Explanations of distress that were non-medical sometimes resulted in women feeling dismissed, and their pain minimized, as can be seen in an encounter Participant 11 recalls having with her mother:

*“I told my mum I was really scared and she said ‘don’t be scared, just pray.’ She meant well*… *I don’t know what I wanted*…. *I think that is often the reaction in my culture, is ‘just pray’ but sometimes that doesn’t cut it”* (Black African).

Women reflected on their parents, and the ways their expectations of themselves influenced whether they sought help. They felt many women of older generations, including their mothers, may have “suffered in silence” and could have benefited from help.

*“I think my mum definitely could have had help with her mental health but quite a lot of the women that I knew, I think, just suffered in silence, which I think is true across the board, actually. I have a lot of South Asian friends and friends from Black families as well, and they, and there’s a common theme where they don’t believe that mental health is really a thing. It’s just a very different approach to it”* (Participant_11, Black African).

Women felt a need to actively shake off their own expectations of themselves, and the expectations of the wider culture–reflecting that the difficulty with reaching out for help had its roots in the ways their parents and the people around them viewed mental health struggles and asking for support.

*”There is a misconception that you need to toughen up. You need to be strong. Like asking for help makes you seem weak. I think there is that misconception amongst my people. Like look at this person, that happened and that happened and they are still standing*… *I think it’s a cultural thing. It’s all about expectations, you are expected to be strong”* (Participant_28, Black African).

### 4.4. Shame, discretion, suspicion, and fear: “*I was scared that someone’s gonna take my baby away”*

Many women believed that trying to keep things hidden, and their suspicion of services might have affected how soon they sought help for their distress. They recalled friends voicing concerns at their being in contact with services: “*I remember a friend going ‘are you still going for counseling? You know they could put that on your file? They could say you are mad”’* (Participant_28, Black African).

Expectations of being able to bear pain, and that it is weak to show emotions interacted with a mis-trust of services which reduced the likelihood of asking for help. Consequently, many women suffered in silence for a long time before seeking help wanting to keep their distress discreet: *“I didn’t talk, but I didn’t hide it either”* (Participant_19, Asian Pakistani). Others deliberately hid involvement with services because of the shame associated with services:

*“I just, I don’t know, I definitely was just embarrassed, I’ve never been one to really talk about feelings, I’ve never, and, you know, being from an Asian culture it’s actually a taboo in Indian culture, it’s a taboo to talk about feelings actually”* (Participant_6, Asian Indian).

*“I had to tell my husband like, please don’t talk to your family about it, my extended family about it. I had to talk to my husband on a level like keep this whole thing quiet if I am going to get treatment and erm keep it on the low”* (Participant_2, Asian Bangladeshi).

Suspicion of services could be seen in the numerous women describing fear that they would lose their baby if they asked for support with their mental health. When asked how one participant felt about being referred to PMHS she said *“I was just more scared that someone’s gonna take my baby away, ’cause I was identified as nuts or something”* (Participant_15, Black Caribbean). The fear that that their baby would be removed was the most common reason women did not reach out for help:

*“I didn’t get help for it because I thought they’re probably going to take my child away from me if I’m honest and I was in a really horrible situation”* (Participant_4, Asian Indian).

### 4.5. Others’ judgement and discrimination leave women isolated: “*Not good enough*”

Many women described how people with mental health problems are labeled as “crazy” and “mad” by the people around them. This had a clear emotional impact, and sometimes a practical impact. For example, not being accepted by in-laws because of their mental illness led women to having fewer supports around them. This made it difficult for women to seek help for their perinatal mental health problems and women were afraid of what their family members would think about them if they found out about their mental health problems.

*“In my ethnic background they don’t really understand it very much. They just think that you’re crazy, that she’s just gone crazy or loopy, you know and that’s the idea they have about it, especially being in a hospital”* (Participant_2, Asian Bangladeshi).

Participants felt that having mental health problems was dismissed, and that they would be judged: *“They would have judged me* (…) *saying ‘she is not good enough to have had kids”’* (Participant_3, Asian Pakistani). Another layer of fear of judgment and stigma appeared linked to migration status, and comparing Bangladeshi people in London and Bangladeshi people in Bangladesh: “*They would also say things like: ‘This is what happens to people who are brought up in Britain”’* (Participant_2, Asian Bangladeshi).

Being discriminated against and verbally attacked by close family members for having mental health problems led women to reduce their contact with services. Participant 13 recounts meeting her mother in-law*: “I met my husband’s mum and I explained to her that I’ve got mental health.* (…) *She kind of used it against me in a, in a sense that*… *she said oh I’m mad like if you’ve got mental health in Jamaica it’s like you’re crazy and mad, so she didn’t accept me as her son’s partner, because I wasn’t good enough.* (…) *It made me feel really bad and I think that’s why I kind of stopped (contact with services) for a bit”* (Participant_13, Black Caribbean).

This had the double effect for some women of leaving them without family support, or support from services: *“It’s just me and my husband at the moment, like we don’t have extended family. They don’t want to know my son*… *she said he could be as mad as me”* (Participant_13, Black Caribbean).

## 5. Theme 2: Hidden and disorganized services impede getting support

Women described not having any idea that there was help available for women experiencing mental health problems in the perinatal period, or what the word perinatal meant, and what PMHS offer. Not knowing that the services existed was described as the biggest barrier to accessing help from services alongside concern that services would take their child away from them. Even when women did reach out for help, after having been referred or because of previous experience of services from an earlier pregnancy (or before pregnancy), it was not always easy to see where to get help from. Many women felt they were left disappointed and in the dark about what was happening. Asking for support but not getting any support damaged fragile trust in services.

### 5.1. Not knowing what support is available: *“I was like, peri what?”*

Almost all of the participants described not knowing that PMHS existed: *“I didn’t actually know that the service existed. It was nice to find out that there was support for women struggling with their mental health”* (Participant_11, Black African). Of the 37 women interviewed for this study, only 5 knew that mental health support services for women in the perinatal period were available, before they accessed care. Even women who had been involved in mental health services for years, and before their pregnancy, and in secondary mental healthcare, were not aware: “*Perinatal is something new, like I didn’t know that before I was referred in my first pregnancy*” (Participant_1, Asian Pakistani).

Others knew that postnatal depression was possible, but not what was available by way of support: *“I’d heard about perinatal depression, but not that there is a team”* (Participant_18, Asian Pakistani). This lack of clarity as to what was available in terms of support made it difficult for women to access help.

*“Like peri what? I literally I was just saying it to my friend, like I’ve never heard of a ‘Perinatal Team’ before. I’ve never heard of it. I asked her if she’s heard of it, and she said no”* (Participant_10, Black Caribbean).

This meant women, even when they realized and admitted to themselves, and to the people around them, that they needed help, did not know where to turn to, resorting to the internet to try and find information: *“I looked it up on Google and the NHS website. And a family nurse and midwife told me more”* (Participant_20, Black African and Black Caribbean).

Even knowing the mental health system did not mean women felt able to navigate getting help. The lack of clarity as to what support was available and how to get it was described by almost all women:

“*Like, who do I need to contact? That’s the point where I was struggling, I was like I don’t know who to talk to because my CPN (community psychiatric nurse) is just a CPN he’s not a perinatal CPN so do I talk to him? Do I reach out?*” (Participant_37, White and Black Caribbean).

Reaching out for help was often met with silence. Women strongly felt that all women should be made aware of PMHS at the start of their pregnancy, irrespective of whether they have known mental health difficulties. Suggestions on how to increase awareness of PMHS included advertisements on billboards, posters at GP surgeries and places such as Women’s Aid, and leaflets distributed by midwifes, at antenatal clinics and at community organizations. The information about the service should go beyond saying it exists, women felt clarity about the different types of support provided was essential to make sure more women from their communities got support when they needed it.

*“I think there should be more advertisements, especially on, maybe on billboards or something about the perinatal team because I think sometimes it is a language and there is a stigma going on, but sometimes Asian families are not aware of the services that you guys provide at all. They think that, no, she’s just going to be stuck in hospital all the time and how will I deal with the other children, and stuff like that”* (Participant_1, Asian Pakistani).

### 5.2. Opaque and inconsistent services get in the way of women who are seeking help: “*I kind of felt I fell through the cracks a little bit”*

Actively seeking help left many feeling abandoned when services did not respond: *“So, I basically just kind of chased up with my doctor and just tried to, yeah, to get him to basically refer me to the perinatal team”* (Participant_24, Black African). Women described waiting and referrals not being followed up: “*I kept on chasing the appointment because I wasn’t getting one through, but I never got one until, I think it was a month before she was born. I needed to like really, really chase it”* (Participant_37, White and Black Caribbean).

Despite chasing, many women found themselves waiting a long time to get any help: *“Well it took a while which was kind of annoying. I wasn’t upset I was just a bit frustrated, because you’re only pregnant for 9 months”* (Participant_27, Black Caribbean). Another participant said *“It was weird because I was aware of becoming unwell and I tried to reach out but just nothing was happening for a while”* (Participant_5, White and Black Caribbean). She went on to say:

*“I think I did reach out to the perinatal team and they said, OK we’ll let them know, never heard anything. I don’t know what happened there so I kind of felt I fell through the cracks a little bit.”*

Not following up on promises broke trust in services which was fragile to begin with and made women feel un-safe: *“I felt kind of abandoned”* (Participant_5, White and Black Caribbean*).* In stark contrast to most women’s sense that PMHS were disorganized, and overly passive, a few women felt PMHS were highly responsive: “*the receptionists are lovely, they pick up straight away, they get back to you”* (Participant_3, Asian Pakistani). The difficulty getting an appointment with the GP was contrasted with the ease of getting in touch with PMHS.

*“Because now at times, it’s very hard to get an appointment. I did one day. Then they said I have to do it online. So, I don’t know how to do it. I’m not very good using online. So, (laughs) I didn’t call back to them again.* (…) *If I didn’t get any help, I stop calling them but in perinatal, if at any time, if I need any help, they try to solve my problem. So, I feel safe. Then next time, I’m feel able to call them and explain my problem because if you get help one time then next you call the same place. If you do not get any help, then you will not get that place another time”* (Participant_31, Asian Bangladeshi).

Services were described as providing support that did not last long enough and appeared inconsistent. Plans were not followed up on, promises and referrals were not always followed through, and women felt that check-ins were not as frequent as was needed: *“She felt like check-ups weren’t done on time, and weren’t as regular as they should be*” (Participant_19, Bangladeshi, interpreter interview). The lack of transparency at the beginning of treatment was a big part of women’s complaints. They were not sure what to expect and left guessing, surprised by the end of support, and often disappointed. This was true of overall support on a macro-level, with the 1-year cut-off of support arriving too soon for many, and on a micro-level for individual sessions ending without warning:

*“The conversation came quite to an end, quite abruptly. Like I was in mid-sentence sometimes. So that was really off putting”* (Participant_23, Black Caribbean).

Conveying lack of interest and unexpected endings damaged trust. Women called for services to be friendlier, more consistent and convey that they were interested and care: essentially elements of standard good practice “being friendlier” (e.g., Participant_37), reading the notes, and being consistent and on time. They felt there should be more pro-active outreach from services, and inquiring several times to make sure women are okay after giving birth. Given the difficulty of sharing that they were struggling, because of fears about their child being removed, women felt more probing was needed to make it seem like services actually cared and had their best interests in mind:

“*She just asked me one day, this lady, she just said, have you been feeling down or anything? And I just said, no, when I had, and that was it, she signed it, OK, no, and then signed off and never came back so I never really saw anybody again and I felt kind of abandoned”* (Participant_23, Black Caribbean).

Women felt that when they were first coming to terms with the idea of needing help, that services appeared to not really want them to disclose distressing feelings. Women felt they needed to be asked more than once for an honest answer to be shared.

Women knowing that perinatal support is available was seen as the most important change needed. Women thought staff should be more transparent about the services available and how to get them, and invest in advertising the details of what help was possible, and that services want to help, and not harm, the mother-baby bond. Many women described it as a problem that getting help relied heavily on women, asking, insisting and chasing help:

*“Services are only helpful if you go to them and you say ‘I need help.’ They won’t help the silently suffering”* (Participant_3, Asian Pakistani).

Another inconsistency was in the provision of interpreters for women and their family members. Some women were informed that interpreters were available, whereas others were never offered an interpreter at all.

## 6. Theme 3: The role of curiosity, kindness, and flexibility in making women feel heard, accepted and supported by clinicians

Whereas theme 2 illustrated a range of difficulties in accessing services, this theme brings together the experiences women shared once they were in services which for many were positive. Women described services listening as having a transformative effect on their lives and sense of self. They felt stronger, re-invigorated with hope, cared for and heard: “*I feel loved and supported and cared for”* (Participant_26, Black African).

### 6.1. Services are nurturing: “*I am a different woman, they gave me my self-respect”*

Women mostly described positive experiences of being in services once they managed to access support: “*According to me, it’s the perfect team. I have no complaints against them*” (Participant_37, White and Black Caribbean). Support was transformative, making them more confident and happier: *“So that is how much they helped me, they gave me*… *encourage, encouraged me and boosted me, and helped with everything”* (Participant_7, Asian Indian). Many women felt strengthened by their contacts with services. Many appreciated that clinicians came to their homes, going so far as to say services felt like family:

“*Knowing that I had no family, they were like my family before I had the baby. So coming home to see me, to make sure I’m ok, I feel loved and supported and cared for. Somebody is there looking out for me and if I wanted someone to chat to about anything that was troubling my mind or disturbing my mind. If I was struggling with anything: school, sleep, emotionally, psychologically, they were there for me at home. Because when they come over, they make sure I’m okay, ask how I’m coping”* (Participant_26, Black African).

Women often contrasted the perinatal team to other services, and sources of support in their lives, singling them out as unique: *“Only the perinatal team. There are the only ones who used to listen to me. Social services didn’t help. I am a different woman, they gave me my self-respect”* (Participant_35, Asian Pakistani, interpreter interview). The same woman said her involvement with services was transformative, and had a long-lasting impact *“When they speak to me, I feel strong. I feel like I’ve actually got someone* (…*). The difference between the ground and the sky–there is an expression in our language, that is how different I feel.”*

Remembering details of their lives, being empathic and showing curiosity were seen as what made services so helpful: *“She always knew, and she remembered every little detail, she remembered my son’s name and she remembered, oh yeah, you were saying about your mum and this, just those things just make you feel like you’re heard”* (Participant_5, White and Black Caribbean). Services became a lifeline for many, a source of relief in the unrelenting pressures they were under:

“*Those weekly sessions, they’re usually something you kind of hold on to* (…*). You can’t wait to kind of get it off your chest”* (Participant_24, Black African).

### 6.2. Respected and treated equally, mostly: “*I’m a human, I’m getting help and people are good”*

Women were asked about whether they felt their ethnicity impacted their experiences of services. Many women said that they were not treated differently or discriminated against based on their ethnicity, culture, or religion. Many women echoed the sentiment that PMHS *“don’t see color or race, they just saw a mother that needs help”* (Participant_15, Black Caribbean). Many said they felt it had no impact, and almost seemed confused by the question *“I don’t think it had an impact.* (…) *I was treated equally* (Participant_1, Asian Pakistani). Such sentiments are echoed below:

*“I–Erm, certainly not in terms of racism, I don’t think. I don’t think they’ve been different to me, because of that”* (Participant_17, Asian Pakistani).

*“Yeah I thought that they’re respecting and they don’t treat anyone differently, regardless of what faith, you know background, regardless of anything. I think that they do understand, because I felt like they were really nice and helpful, like no one discriminated in any way* (Participant_29, Asian Bangladeshi).

However, one woman described her clinicians not thinking she needed help, which she wondered about and wondered whether her being Black played a part:

*“So, for example, they said that I didn’t need the help to start with and I’m thinking well why is that? Because they needed the help the first time? And it does play on your mind a little bit, ‘is it because I’m Black why I’m not being offered this help?’ or ‘is it because of this, is it because I’m a Rasta, is it?’ And you don’t want to make too much of a deal about it but it does flicker through your mind a lot of the times”* (Participant_22, Black African).

Perhaps indicative of tellingly low expectations, some women appeared to be surprised that they were not treated badly, or discriminated against in services: *“I think it was really good actually especially being Asian, I think sometimes I worry, thinking ‘will people be nice to me?,’ ‘will people treat me differently?’ because you know, I get, I don’t know, maybe I’m, it’s just me but I just get really worried because obviously, I moved to this country when I was 18 and I really struggled to learn the language, to blend in with people. And, so I think it was my, initially, I get really worried especially like (DOCTOR’S NAME) sounds so strict. But meeting somebody who’s getting a warm welcome and just the first impression really makes a difference”* (Participant_3, Asian Pakistani).

### 6.3. Flexibility and curiosity in staff inspire feeling accepted and respected: *“Yes, she understood me, because she asked questions”*

Several participants described services’ flexibility in relation to their culture as helping them feel accepted and respected. This was shown by asking questions, and being flexible: “*When I was fasting, Ramadan, they basically valued that, about the fasting, because I said I wanted to fast”* (Participant_1, Asian Pakistani). They also said the provision of interpreting services was important: “*Like by having a translator for my husband. That was really good and also them being loving and caring as well and supportive, really supportive, which helped”* (Participant_1, Asian Pakistani).

Women felt services accepted customs and traditions that they wanted to observe, and this curiosity, open-mindedness and flexibility was seen as vital: they did not feel the staff needed to know everything about their culture but felt respect and interest from staff members who inquired. Asking questions, without making assumptions, was seen as important. Curiosity was underlined as essential in maintaining a sense of being accepted and respected, and crucially, in feeling understood. When asked whether she felt services understood her, one woman said: “*yes, they understood me.* (…) *I told them about my religion, they asked me about my religion and then I told them. They were OK with it”* (Participant_19, Asian Bangladeshi, interpreter interview). She went on to explain that asking, and curiosity was what made her feel understood. This was echoed by another woman who when prompted to explain why she felt supported said “*She (the clinician) asked me*… *she delved into cultural things”* (Participant_10, Black Caribbean).

Women described that everyone’s needs would be different when it comes to the meeting points of mental healthcare and culture and ethnic background: *“Obviously it would depend on individual circumstances wouldn’t it because Joe Blogs next, a Black person next door to me is going to have different needs to what I have”* (Participant_22, Black African).

Women provided examples of how their religion, or cultural practices had been considered during their care. This included having a separate room at the mother and baby unit to practice religious beliefs and offering to arrange an Imam to visit the mother and baby unit to provide reassurance that it was not necessary to fast during Ramadan. One woman explained that PMHS had prior understanding and were supportive of having a naming ceremony 7 days after birth of her child or wearing a wrapper around the ward. For one woman, who wanted to continue fasting, it was arranged for her medications to be delivered in the late evening and early morning, as described below:

*“They didn’t force me to stop fasting and stuff.* (…) *So they used to have someone in the late evening, after I opened my fast, to come and give my medication to take, and one of them would come in the morning before the Sehar time”* (Participant_32, Asian Bangladeshi).

For a few women, PMHS referred them to other services that could provide additional support tailored to meet their specific cultural needs. This included counseling that was specific to women from Islamic backgrounds and was facilitated by professionals from the same background who had a better understanding of their culture.

In addition, many women felt that PMHS understood the stigma and taboo around mental health problems that exists in most cultures. Staff were aware that mental health is not always something that is talked about openly with family members and respected women’s wishes for their mental health to not be discussed with others during home visits. The stigma around mental health was also addressed during appointments. Staff explored wider family member’s understanding of women’s mental health problems and often asked women about their support systems outside of services. This helped women feel understood and supported, as described below:

*“My doctor used to always ask me does your Mum understand you or does culture get in the way? So, I am sure she knows that culture is a barrier to like parents understanding young children or children that are suffering mental health problems. So, I think that she was more supportive cos everything I spoke about I wouldn’t be able to speak to my parents about.* (…) *I felt she understood cos for her to pick that up and ask me was a big thing”* (Participant_30, Asian Bangladeshi).

Other women reported that PMHS did not consider their culture throughout their care, however, whether this was important varied amongst women. For some women, it did not matter that ethnicity was not explored during their appointments as it was not deemed relevant to their mental health, life or care within services. However, other women felt that their culture was not understood because it was never explored. These women felt it would have been beneficial to speak about the impact that their culture and upbringing had had on them, as well as speaking about parenting and childrearing practices that are important to them:

*“Well, it (culture) wasn’t asked about and there were certain things where I said, no, I’m just not going to mention it because I know what they’re going to say like co-sleeping and they kept on saying don’t co sleep, don’t co sleep because your medication might make you tired, blah, blah, blah. And I’m just like, well that’s what we do in Bermuda really and it’s part of the bonding process for me and rather than telling people, no don’t do this, maybe say, OK, this is a way we can try to do it safely because I only found out how to do it safely by researching on the internet”* (Participant_5, White and Black Caribbean).

Not trusting services was important as it reduced how forthcoming women were with their actual experiences, and disclosure of distress, and risk. One woman said “*After a while, I kind of like developed a trust* (…*.) Cause at first, I kind of like hid how I was feeling.”*

## 7. Theme 4: A shared cultural background may support or hinder trust and rapport

### 7.1. Sharing ethnic background with a clinician is unimportant, and even unhelpful: “*It doesn’t make a difference”*

Many women felt their practitioners’ ethnic background was not important, but their behavior was what mattered: *“No it doesn’t make a difference where the doctor is from”* (Participant_36, Asian Bangladeshi, interpreter interview). Several women felt what mattered was how kind, curious their clinician was rather than where they came from: *“I think that it would have been the same regardless, I think, yeah.”* (Participant_5, White and Black Caribbean).

Shared ethnicity was also described as acting as a barrier, in certain cases, where they felt the shared culture would come with a judgement. Women also described difficulties related to sharing an ethnic background with a clinician.

*“Yeah, my therapist was Indian, she was the same as me, she was Punjabi, and initially I was really embarrassed, I was thinking, oh no, she’s even got the same name as my husband, this was a nightmare for me.* (…) *That she might know my in laws, she might know my, you know, my husband. That was a nightmare for me”* (Participant_6, Asian Indian).

Women described listening, feeling understood as mattering most, and that that was not related to where the clinician was from. Some women went so far as saying they felt less well understood when seen by people that shared their culture because of what they believed the clinician would be thinking and saying about them. One woman said she felt staff on the ward that were not from her specific ethnic background were more understanding: *“I thought they understood me better than my own Bengali people.”*

*“She has always listened. It wasn’t like, one way just making decision of the treatment, I was able to make my treatment plan, everything, and she was a good listener and she.* (…) *I was given opportunity to share my feeling better and for them to understanding and, which is also a relief. It was very important that you do get a chance to share how you feel, and I was given the chance by the team that I was under. That is what mattered most”* (Participant_32, Asian Bangladeshi).

### 7.2. Clinicians and patients sharing a cultural background is a shortcut to rapport

Several women described having healthcare practitioners from their cultural backgrounds as helping them feel understood. The importance of visual representation within services was emphasized: *“I would like to see more practitioners of color. More diversity. I think it makes people feel more comfortable in terms of referral and stuff like that and to be able to speak to someone who reflects their background culturally”* (Participant_23, Black Caribbean).

Women reported thinking it was easier to open up to healthcare professionals who were from the same background, or a similar background, and harder to open up to White healthcare professionals. Women held back from mentioning certain things, anticipating that they would not be understood or well received, as described below:

*“I’ve noticed myself most of the practitioners you see are White and they’re British, and they don’t have really the same experience. So, you are wary* (…) *you think to yourself oh I might hold back on this, because culturally, I’m not sure how this is going to go down”* (Participant_23, Black Caribbean).

Having a shared background was described as helpful in expressing themselves, that would not require as much explanation.

*“There’s certain, yeah, just terms, proverbs.* (…) *When you say it to somebody else, you might have to explain it if they’re not from the culture. Whereas if they are*…*They’re like, yeah, I, I know what, I know exactly what you mean”* (Participant_24, Black Caribbean).

This ease in expressing thoughts and feelings helped women feel that they were understood by their clinicians: *“To be quite honest*… *I think (NURSE’S NAME) she had a bit of my same background, like from Africa so she understood what I’ve been through, what I went through”* (Participant_26, Black African). Women saw certain things as impossible to learn from services, and certain experiences being needed to be understood*: “they’re trained in [mental health] but they’re not trained in Black culture. They’re not experts in that”* (Participant_12, Black Caribbean).

One woman described a change in staff that resulted in her replacement clinician being White, which she thought impacted how well the clinician understood her*: “I feel like I had a bit of a culture clash*… ’*cause erm what happened was my care coordinator took time off after I gave birth or something*… *and then I had, I got replaced with someone else that was like, she was African. So it’s different culture from Caribbean. (*…*) Then I had my nurse who was coming in, she was*… *She was White, and like I felt like I had coped better with the person that was Black”* (Participant_15, Black Caribbean).

## 8. Discussion

### 8.1. Key findings

Four themes reflected the experiences of Black and South Asian women in accessing PMHS and the care they received. The first theme captures the barriers they experienced that came from within themselves, the people around them and differences in the ways in which they understood distress and services’ (primarily medical) understanding of distress. The second theme captures the practical issues that act as barriers to receiving and engaging with care. The third theme captures the predominantly positive experiences of women once they were receiving support from outpatient services and inpatient services. The fourth theme describes views on what is important in building a rapport with clinicians working in PMHS, and the divided views women held on whether having the same ethnic background as their clinicians was helpful to build rapport, not important, or a barrier to building rapport. Internal and external social and cultural factors play a part in slowing or stopping help-seeking and accessing services for Black and South Asian women, however, many women in this sample overcame these barriers and were met with service-level organizational barriers that slowed their ability to benefit from services. Women described difficulty in getting support, and that the support was transformative when they did manage to access it. Important threads that show up across different women’s accounts, and appear important to access care and remain engaged with clinical services, are described below in relation to the broader literature. The suggestions for how to improve services broadly fall within a need for greater connection and need for better awareness, bidirectionally between services and the populations they are trying to serve. This awareness is firstly that PMHS exist; secondly, awareness of *what* those services might look like in practice (e.g., that outpatient community support exists as well as inpatient admission). Thirdly, the findings point to a need for clear communication and expectation-setting from the outset of involvement with services.

### 8.2. Comparison with the literature and recommendations for further research

The mistrust of services is an important finding and should inform campaigns attempting to better support women. It is noteworthy that the fear of losing their baby and suspicion of services is threaded throughout the themes: a reflection of how omnipresent the distrust and fear was for participants. Women described this as a key factor that prevented them from, or slowed, their reaching out to services. The findings should also be considered within the context of the role institutions have historically played in propagating systematic racism (43). A recent scoping review found people with marginalized identities are more likely to be wary of institutions (44). Research in predominantly White women in the UK has also found a fear that services would remove their children and distrust (45–47). It may be that there are compiling, and intersecting factors at play in this sample that make it less likely Black and South Asian women will disclose mental distress in the perinatal period: women’s concern about being able to stay with their baby, and a longstanding lack of trust in institutions of power. It appears reassurance of services’ role, and a gradual building of fragile trust may be particularly important for women from marginalized and minoritized groups, particularly if they are in intersecting groups that are marginalized as is the case in this sample who have mental ill-health (44). These intersecting identities may also increase vulnerability; for example, research in Scotland has found that Pakistani, Indian, African, and Caribbean individuals identified experiences of racism as having an impact on psychological and physical health (48). More work is needed to understand specifically how this may play out in mental health services, as much of the existing research reviewed is in the context of physical healthcare (44).

This study found that stigma related to mental illness was an important internal barrier. Interestingly, a survey of Australian mothers did not find that stigma was a barrier for women accessing care, but rather that lack of awareness prevented seeking help (49). It may be that stigma is a more significant barrier in the group of women interviewed in the present study, and interacts with a lack of knowledge to make women from ethnic minorities less likely to access help. This may be further compounded by clashing understandings of mental distress between services and patients/their families. Understandings of the world, what it means to be human and what mental distress is, are all learned in large part from our close family, and culture. There is support for the clashing views of mental distress playing a part in help-seeking behavior. In a study of ninety-four British Muslims of South Asian origin, shame, or “izzat” and biological beliefs about the causes of mental health difficulties and shame/izzat were negatively related to the intention to seek help. The researchers studied “acculturation” and found lower scores predicted lesser intent to access psychological services, whereas higher levels of “acculturation” and education predicted greater intention (50). More research, perhaps medical ethnographic studies, should examine these differences in understandings of distress so that services can learn how to describe patients’ experiences in a way that is more inclusive and flexible.

The finding that many women had positive experiences in services should be considered within the context that all of the women interviewed had used clinical services and were recruited through them. This could have biased the results toward the positive, because women who did not like or benefit from services may have disengaged from services and/or their clinicians might have not put them forward for the study. There could therefore have been bias introduced, by having certain voices missed. The experiences of South Asian and Black women who did not access services, despite experiencing perinatal mental distress, are presented elsewhere (51). It is encouraging that women interviewed for the present study described feeling accepted and respected. Many felt services were helpful, even transformative. These findings suggest PMHS are able to deliver patient-centered mental healthcare, and perhaps that recent investments in services and staff training seem to have contributed to services’ strengths, including a focus on providing high quality support inclusively to a diverse range of people (52). Further research should explore whether this is the same across inpatient and outpatient services, or whether there are differences between women who were admitted to Mother and Baby Units or those admitted to adult wards where they would have been separated from their babies, or not admitted. It is possible that having an admission and more time to get to know staff and forge a relationship would be associated with a better experience of care–or the opposite may be true, as reports have described psychiatric admissions as traumatizing in themselves, in particular involuntary admissions (22). An important novel finding of this study is in detailing what made these good experiences described by women so positive. Women said empathy, kindness and clinicians seeming to remember and have an interest in their lives, helped them feel heard. Although empathy, curiosity and reading notes should be existing parts of standard good practice, it is important to explore these to highlight *why* they are part of good practice and the impact they can have. Often in accessibility research, the focus is on what is wrong. Although this is of course essential, increasing the likelihood of positive experiences of care is possible only if attention is paid to them. The specific elements described (such as trust, support, curiosity) fit with existing research on the therapeutic relationship, and on non-specific factors in psychotherapy (53–56). The transformative impact of these basic tenets of good practice can be seen in this study’s findings, and serves as important reminder for clinicians in services.

Low rates of service use in Black and South Asian women have been presumed in the literature [e.g., (15)] to be related to women being referred but not accepting referrals, and not engaging with services. The finding that women chased appointments and fought to receive care fits with Jankovic et al. (4) study which showed that low engagement was not a credible explanation alone for low uptake of care. Although there are barriers to help-seeking (theme 1), these alone cannot account for lower levels of care received, as many women chased appointments and did not access care despite seeking help (4). A systematic review suggested complex interlinked factors related to resource inadequacies, service fragmentation and unclear policies as barriers to all women accessing support in the perinatal period (47). New avenues to address these barriers (such as making referral pathways more transparent) have been highlighted in the present paper, so that service organizers and clinicians can take action.

Recruitment for this study was carried out primarily in East London and Birmingham. These areas have a high proportion of women from South Asian and Black ethnicities (57). This means that participants’ views may have been different had they been recruited from areas where there were fewer women who looked like them and were from the same background: both other patients and clinicians providing care were ethnically diverse. This is relevant for several reasons. Experiences of discrimination and racism may be more likely when living in a less ethnically diverse area, and discrimination has been found to be associated with worse mental health (58). In addition being in an area that has more people from the country of origin is protective for mental health (the so-called “ethnic density effect”) (59). This also means many of the women who were recruited in this study had clinicians who were of the same background as them. It could have an important impact on how they experienced care. A study investigating women who did not engage or disengaged from PMHS Jovanović et al., (51) found that many women were the only person of their ethnic group in services. This was less likely to be the case in the participants recruited to this study. It may be that theme four’s findings would not generalize to other populations with less ethnically diverse staff. The complexity of feelings around the background of the health professionals can be seen in other literature. A systematic review and meta-ethnography of the experiences of South Asian patients in mental health found a similar finding, that people felt they were stuck in a dilemma of mistrusting both White and Asian professionals (15).

Several factors should be considered alongside women’s reports that they were not treated differently or discriminated against because of their ethnicity. Firstly, it is possible women had low expectations because of past experiences and internalized expectation of mistreatment and discrimination. For example, not being forced to abandon a religious practice, which was described with gratitude by several women, might be considered to be a low bar to have set. Perhaps women have not felt even that (relatively low) level of acceptance of their faith in other areas of their life, in particular Islamic women in the current climate of Islamophobia on the rise in Europe and the US (60). Several women described surprise at not being treated badly, which lends credence to this interpretation. It may be that subtle experiences of discriminatory treatment are therefore not consciously picked up on by women because they have grown to expect they are normal (61, 62). It may also be difficult for women to share experiences of discrimination with interviewers (many of whom were White) that they had only just met (see Supplementary material 3 for details on interviewers). The finding that women were not discriminated against contrasts with other relevant literature, including a recent survey which found that 65% of Black respondents had experienced prejudice from doctors and other staff in healthcare (63). Furthermore, it also contradicts evidence from a rapid review which found evidence of stereotyping, disrespect, discrimination, and cultural insensitivity across maternity services (64). This led to women from ethnic minority groups feeling “‘othered,’ unwelcome, and poorly cared for” (64).

The findings should also be considered in light of the context recruitment took place in. Recruitment began before the first lockdown and continued throughout the UK lockdowns. This momentous context for women to have been experiencing PMHS might have had an impact on their experiences. Some women were describing experiences of health services before the pandemic and some during, but most interviews took place after the first lockdown. In the context of seeing health services and healthcare workers as heroes in the news and hearing accounts of how difficult it was to get support, they may have been more grateful and appreciative of the support offered by PMHS than they would have been without the context of a large pandemic acting as a unifying and relativizing force. Movements such as the weekly “Clapping for Carers” which started in March 2020 in the UK reflected and boosted goodwill for NHS frontline workers (65). The context women experienced care in was part of the topic guide, however, no trends between groups (people who experienced care before, during or after the national lockdowns) were observed.

### 8.3. Recommendations for clinical practice

It is important to consider the finding that services are disorganized (theme 2) in the current context of PMHS in the UK. There has been a large expansion and investment in services since 2016, and thus a rapid change in the services that are available (52). This comes with great advantages for the women being served by services, however, the newness also means that there may be a particular lack of clarity about the referral processes. This confusion is shared by clinicians in many cases (66).

The implications of the study’s findings relate to different levels of service organization: from the commissioning of services, to the way communication about these services is conveyed to the general population, and the day-to-day organization of who answers the phone/is in reception when someone is in distress and asking for help. Although organizations may seem immovable and too complex to change, there are key areas highlighted by these women’s experiences that deserve investment in understanding and focused action so that women are no longer left suffering in silence. In a first step, the findings indicate there is a need for public health policy campaigns to advertise and raise awareness that PMHS exist, and what types of support they offer. Further, more resources being invested in PMHS would allow for the threshold for referrals to be lowered. This would prevent women from feeling dismissed in their distress and prevent mild distress from becoming more severe. We are aware that this recommendation may seem out of line with the reasons that PMHS were initially targeted: namely to address moderate to severe conditions. However, with the 5 Year Forward plan, and recent investments, services have been changing to be more inclusive, with a view of intervening earlier and focusing on prevention (52). The findings indicate there is a need for more work to proactively identify women who are suffering and to offer them support, as there are so many layered barriers to getting help. We suggest working with community organizations to address stigma, improve links between primary and secondary care services to avoid women falling between the cracks, and providing assistance with childcare so that it is possible for women to attend appointments. Furthermore, there should be more immediate appointments that are easier to access. There should also be better implementation of identified care pathways. Implementation research should examine the practical barriers that might, and currently do, get in the way of these recommendations being enacted. These recommendations fall in line with studies aiming to improve access to PMHS for all women in the UK as well as studies conducted in other settings including Australia (49, 67, 68). A cross-sectional survey of *n* = 218 Australian women found that over one third of participants were not knowledgeable about mental illness in the perinatal period. Of the mothers asked about their mental health, only one third were offered a referral. Ayres and colleagues concluded that universal screening, faster appointments and the provision of support with childcare is needed (49). Existing initiatives aim to enact these principles outlined in this paper and in the PAAM study’s recommendations for clinical practice (elaborated on below), such as (this study was part of a larger NIHR-funded project, the PAAM project, which aimed to improve access to perinatal mental health services in ethnic minority women) the PATH project which aims to design, deliver, and implement new, durable PMHS. PATH, funded by the European Commission, aims to prepare parents pre-birth for their new role and help them avoid perinatal mental illness. The first output of the project is a multimedia campaign to raise awareness and de-stigmatize perinatal mental illness (69).

The PAAM project included the present study of women (*n* = 37) who accessed care, and other quantitative and qualitative studies examining other perspectives. Based on the condensed findings across all these studies - including views of women who did not access care (51), healthcare professionals (66), family members’ (70) *inter alia*–recommendations for practice were made by an Expert Reference Group comprised of women with a lived experience of perinatal mental illness, psychiatrists, midwives, nurses, third sector workers, commissioners, and researchers all from a diverse set of ethnic backgrounds including South Asian and Black ethnic backgrounds. These recommendations will be made publicly available (please contact the corresponding author or check the study’s Twitter account for updates @PAAM_Study).

Women’s experiences of services and suggestions for improvement of services ranged widely, with many unique responses. Simply because most individuals made different suggestions does not render their recommendations less important but indicates that an individualized approach should be taken and that no one should assume that they know what women think, want and feel based on particular facets of their identity, such as their ethnicity. The most commonly suggested avenues for improvement in access to and acceptability of services was to advertise the existence of services and set clear expectations as to what type, and amount, of support could be expected. Most suggestions were practical and concerned the functioning of services in an efficient way, the quality of care provided and clinicians being respectful, showing up on time and following up on promises. Building trust, listening, patients feeling heard and not abandoned were detailed as important priorities for services going forward, which fits with findings that minoritized groups have lower trust in services (47). All of these findings fit with existing research on the therapeutic relationship and the importance of the “non-specific” or “common” factors in producing effects on mental health outcomes (52, 71, 72).

The aim of this study was to obtain views from women who are most likely to not receive care they need, not to compare between different ethnic minorities’ experiences. Furthermore, the small sample sizes within subgroups and the qualitative approach would mean attempting such a comparison would not yield meaningful findings. No differences between ethnic groups could be considered in this analysis, but it is important to note that there was little homogeneity, and women’s individual experiences varied widely. Studies should examine in much more specific groups whether, and how, different local and culture-specific views of mental health, healing and illness impact women’s experiences and behavior.

### 8.4. Strengths

This study provides a novel, in-depth, pragmatic and clinically-useful view of Black women and South Asian women’s experiences of PMHS. It shines light on ways services are currently supporting women. Furthermore, from the perspective of these groups that are underserved, this study provides elements of service organization and provision that should be targeted to redress the inequities apparent in service-use. The specific focus on access and engagement is an important strength as it addresses an evidence gap that was highlighted by Jankovic et al. (4). Building on their finding that access to services is problematic, rather than exclusively *engagement* with services, this study offers possible explanations and hypotheses for future research to explore.

A second important strength is that this study included interviewees who could not speak English fluently and are often left out of care and voiceless in research (73). Conducting interviews with an interpreter means these often-stifled and ignored voices are represented in these data.

A third strength is related to a limitation of the study. The study was initially intended to happen face-to-face, however, the COVID-19 pandemic meant this was changed to become an online interview. The rapport between interviewer and participant may have been weakened by reduced ability to pick up on non-verbal cues. Women were also possibly unable to find private spaces in their homes to open up about these difficult topics, which may have made the information shared on difficult topics more careful, and perhaps less honest. However, a strength came from this: women who might not have been interviewed in person might have been able to take part thanks to the flexibility afforded by online interviews.

### 8.5. Limitations

There were limitations to this study that should be held in mind when considering the findings. All of the participants were engaged with PMHS: they received support from services. The finding for example, that women did not feel discriminated against because of their ethnicity must be considered with this in mind. Evidence from rapid evidence reviews for the NHS Race and Health Observatory point to people from ethnic minority backgrounds being less likely to be referred for specialist treatment, and more likely to experience discrimination at the hands of healthcare providers (64). Women who did experience discrimination at the hands of services are less likely to stay in touch, and may disengage from services, and therefore less likely to end up in this study.

A second limitation comes from the interviewers working on the study: none of the interviewers were Black, and most were White British (see Supplementary material 3 for reflexivity and demographic details of interviewers and analysis team). This means all of the Black women interviewed were by definition not interviewed by someone who had experience of being racialized as Black. It is possible that the findings, in particular those about differential treatment and experiences of discrimination would have been easier to share with interviewers who shared their racially marginalized identity (74). Extensive work has explored the role of researcher positioning, and although it is difficult to study, it appears that sharing is significantly influenced by who one is sharing with (75–77). This is a limitation of the study and means the findings should be interpreted with caution: it is possible, and even likely, that when discussing sensitive topics such as racism, discrimination, culture and belonging, that the person asking the questions plays a part in how participants answer. As the person interviewing in this study appeared either White, or South Asian, their ethnicity may have played a role in how able participants felt to disclose true experiences, which is what has been found in previous research (74).

A third limitation is the grouping of several very different ethnic group belongings in one study. Black and South Asian women were the focus of this study because they are most vulnerable to not accessing care, and more likely to be admitted involuntarily under the Mental Health Act (4). This vulnerability that is shared across ethnic groups does not necessarily mean the problem is related to ethnicity, or that the solution to improving access lies in specific features related to culture or ethnicity. If the important feature impacting the inequality is culture, then a study focused on one specific culture’s understandings of mental health is needed, rather than combining across very different groups. The findings indicate very different experiences within women who identify within the same ethnic category on the British census, as well as variation across ethnic groups. Studying several groups together risks masking the most marginalized, and although the effort was to redress inequalities, there is a risk of further obscuring what is happening for the most marginalized in grouping these women together (78).

Future research should focus specifically on one single specific subset, and inductively, in a participant-led way, allow barriers and solutions to be explored. There is a history of different minority groups banding together, and the commonalities across very different marginalized communities being highlighted, and learnings from one group being applied to another. For example, striking miners and gay rights activists in the 1980s were two groups who, though different, were similar in their being marginalized, and decided to band together in solidarity to support each other’s plights (79). This study similarly studies numerous distinct and different groups who might share little other than being minorities, and marginalized, and both having lower access to PMHS (4). It is important to note these differences in understandings of distress, practices and varying degrees of discrimination and marginalization they experience. This study can act as a first step for researchers to focus in greater detail on specific subgroups, in specific locations so that the nuances of individuals’ contexts and socio-political history can be considered. Quantitative data from the British census points to different outcomes across a range of metrics across different minorities. The most recent census shows, for example, that outcomes are worse in Pakistani and Bangladeshi women than in Indian women (8). Further research should explore how mistrust of services impacts individuals in specific groups in order to find ways of increasing the chances women will get support when they need it.

A fourth limitation comes from the complexity of asking about culture and ethnicity. In the interviews, ethnicity and culture were conflated, and used either in tandem or interchangeably (e.g., often a question was phrased ‘do you think your ethnicity or culture made a difference to the care you received?’). People may understand a variety of different things from those words, which were not defined or explained at the start of interviews. It may be that some women answered the question thinking about “ethnicity,” rather than culture, or vice versa, and it may be that women understood these concepts in different ways.

No records were kept of the number of people who did not participate and reasons for non-participation. Some data on this might indicate how many women did not take part because of a mis-trust, or dislike of services, which may have skewed the findings reported. In addition, data checking was not conducted because it was infeasible given the interruptions to the study because of COVID-19 (80, 81). These are limitations of the current study.

A further limitation to the generalizability of the findings comes from the context of the COVID-19 pandemic. There is published literature indicating that during the COVID-19 pandemic/lockdowns, women in the perinatal period experienced more anxiety and depression (82). They were also experiencing more barriers to accessing services and they were more likely to access services remotely (83). For these reasons, women’s experiences of services were likely to be different compared to in the pre-pandemic period.

There are a lot of hidden views and unspoken experiences on this topic, in particular in women who do not speak English as a first language and who migrated to the UK (84, 85). We made big efforts to make sure that the women who are usually left out of research, and care, were heard. Although it is a strength that this study included women who did not speak English, there were difficulties that are invited by conducting interviews with interpreters. Occasionally the women spoke for what seemed like a lot longer than the interpreter spoke, suggesting the possibility that the interpreter was simplifying what she was saying. This intermediary, the interpreter, might not have known which elements of what the women said were important for the study and there could have been important nuances lost. It is a weakness that the analyzing researcher could not understand any of the words women spoke in their own language. To mitigate the risk of this, future research should explore these questions in greater depth, in women’s own languages, by employing researchers, who know the aims of the study and speak the language fluently. These researchers should be involved in the interviewing process, the analysis and the write up of the findings.

## 9. Conclusion

Experiences of care and how easy it was to access help varied greatly. All women felt that knowing support was available, and what that support might look like, would have helped them seek and access help sooner. Broadly positive, and even transformative experiences of being under PMHS, were attributed to feeling understood, heard and validated. This was made possible by clinicians’ curiosity, kindness, flexibility, and by building trust. Women described chasing appointments, having to ask repeatedly for help, and wishing they had been asked more often about their mood. Finally, women having benefited from services should be underlined as an important finding. The coalescence of factors influencing help-seeking behavior mean multifaceted interventions may be most effective as simply targeting one factor (for example, the stigma attached to mental health) may not be sufficient to result in bringing women the support they need.

## Data availability statement

The datasets presented in this article cannot be made available without risking breaking the anonymity of participants. Requests to access the datasets should be directed to the corresponding author.

## Ethics statement

This study which involved human participants was reviewed and approved by the Health Research Authority via London–Queen Square Research Ethics Committee (19/LO/1830). The participants provided their informed consent to participate in this study. All participants provided written informed consent for the publication of quotations included in this article.

## Author contributions

MC: formal analysis and writing–original draft. KCP: investigation, writing–review and editing, formal analysis, project administration, and data curation. SB and AS: investigation, formal analysis, and writing–review and editing. JJ: conceptualization, funding acquisition, methodology, writing–review and editing, and supervision. HKS: conceptualization and methodology. RM and AC: supervision and writing–review and editing. KB: analysis and writing–review and editing. SP: funding acquisition, conceptualization, resources, and writing–review and editing. NJ: conceptualization, methodology, supervision, and writing–review and editing. All authors contributed to the article and approved the submitted version.

## Footnotes

1 The number of diagnoses outnumbers the participants because some participants had more than one diagnosis.
