# Supplementary material for: Exploring Black and South Asian women’s experiences of help-seeking and engagement in perinatal mental health services in the UK
Source: Front Psychiatry. 2023 Apr 3;14:1119998. doi: 10.3389/fpsyt.2023.1119998 (PMC10109459; doi:10.3389/fpsyt.2023.1119998)
Supplement: Supplementary file 1 [file Data_Sheet_1.docx]

# Supplementary Material

## Supplementary Material 1.

Consolidated criteria for reporting qualitative research (COREQ) Checklist

*Domain 1: Research team and reflexivity*

Personal characteristics

| Item | Description | Page |
| --- | --- | --- |
| 1. Interviewer/facilitator | Which author/s conducted the interview or focus group? | 4 |
| 2. Credentials | What were the researcher’s credentials? E.g. PhD, MD | See below, Supplementary Material (SM) 3. |
| 3. Occupation | What was their occupation at the time of the study? | Research assistants, lived experience researchers and clinical studies officer, page 4 of manuscript. |
| 4. Gender | Was the researcher male or female? | All female, 4 |
| 5. Experience and training | What experience or training did the researcher have? | See below, page 8 of Supplementary Material 3. |

Relationship with participants

| Item | Description | Page |
| --- | --- | --- |
| 6. Relationship established | Was a relationship established prior to study commencement? | No, specified on page 5 of manuscript. |
| 7. Participant knowledge of the  interviewer | What did the participants know about the researcher? e.g. personal goals, reasons for doing the research | page 4, 5. |
| 8. Interviewer characteristics | What characteristics were reported about the interviewer/facilitator? e.g. Bias, assumptions, reasons and interests in the research topic | Supplementary Material 3. |

*Domain 2: study design*

Theoretical framework

| Item | Description | Page |
| --- | --- | --- |
| 9. Methodological orientation and  Theory | What methodological orientation was stated to underpin the study? e.g. grounded theory,  discourse analysis, ethnography, phenomenology, content analysis | 5 |

Participant selection

| Item | Description | Page |
| --- | --- | --- |
| 10. Sampling | How were participants selected? e.g. purposive, convenience, consecutive, snowball | 4 |
| 11. Method of approach | How were participants approached? e.g. face-to-face, telephone, mail, email | 5 |
| 12. Sample size | How many participants were in the study? | 4 |
| 13. Non-participation | How many people refused to participate or dropped out? Reasons? | 4 |

Setting

| Item | Description | Page |
| --- | --- | --- |
| 14. Setting of data collection | Where was the data collected? e.g. home, clinic, workplace | 4 |
| 15. Presence of non-participants | Was anyone else present besides the participants and researchers? | 4 |
| 16. Description of sample | What are the important characteristics of the sample? e.g. demographic data, date | 6 |

Data Collection

| Item | Description | Page |
| --- | --- | --- |
| 17. Interview guide | Were questions, prompts, guides provided by the authors? Was it pilot tested? | SM 3 |
| 18. Repeat interviews | Were repeat interviews carried out? If yes, how many? | 5 |
| 19. Audio/visual recording | Did the research use audio or visual recording to collect the data? | 5 |
| 20. Field notes | Were field notes made during and/or after the interview or focus group? | SM3 |
| 21. Duration | What was the duration of the interviews or focus group? | 6 |
| 22. Data saturation | Was data saturation discussed? | 5 |
| 23. Transcripts returned | Were transcripts returned to participants for comment and/or correction? | 25 |

*Domain 3: analysis and findings*

Data analysis

| Item | Description | Page(s) |
| --- | --- | --- |
| 24. Number of data coders | How many data coders coded the data? | 5 |
| 25. Description of the coding tree | Did authors provide a description of the coding tree? | 5 |
| 26. Derivation of themes | Were themes identified in advance or derived from the data? | 5 |
| 27. Software | What software, if applicable, was used to manage the data? | 5 |
| 28. Participant checking | Did participants provide feedback on the findings? | 25 |

Reporting

| 29. Quotations presented | Were participant quotations presented to illustrate the themes / findings? Was each  quotation identified? e.g. participant number | 6-19 |
| --- | --- | --- |
| 30. Data and findings consistent | Was there consistency between the data presented and the findings? | 6-19 |
| 31. Clarity of major themes | Were major themes clearly presented in the findings? | 6-19 |
| 32. Clarity of minor themes | Is there a description of diverse cases or discussion of minor themes? | 6-19, Table 1, Figure 1. |

## Supplementary Material 2. Topic guide

**Topic guide: Qualitative interviews for PAAM (WP3):**

**Mums with PMI that accessed PMHS**

1. **Introduction**

- Introduce self
- Explain nature and purpose of the research (e.g. this interview is about exploring your views of your perinatal mental health illness, your experience of using Perinatal Mental Health services and how we can make these better and easier to use for mums from ethnic minorities)
- Provide assurances about confidentiality and no link to current treatment/care (e.g. your name and information will only be known by the researchers and will not be revealed to anyone else)
- Explain what happens to data collected – recording, transcribing, analysis, anonymity (all potentially identifying information will be removed), audio recordings will be destroyed once data analysis is complete, check how they would like to be referred to during recording
- Introduce tape recorder
- Explain how interview works- interviewer will not say very much and will tend to ask questions, all views important, no right or wrong answers, looking for a range of views
- To say if they are feeling stressed/uncomfortable. Do not have to answer a question or can change topic if needed. The interview can be paused at any time if you require a break.
- Mobile phones off or on silent
- Invite any questions

*Note for interviewer:*

*As far as possible make sure you probe and ask for specific examples. So, if the participant states that ‘needs specific to their culture were not met’ ask the participant to provide specific examples of this.*

1. **Background**

**🡪 *Ask participant about themselves (e.g. how many children they have, who they live with, do they work, availability of close friends and family members as support) and about their experience of pregnancy/ies and postnatal period,***

*e.g. tell us a bit about yourself and your experience of pregnancy and after the baby was born*

*By the end of this section the interviewer should have a clear understanding of the mother’s mental health difficulties, including the origin, early signs, development and impacts on mother and child as well as the wider family if relevant.*

*The purpose of this section is to obtain a clear understanding of the mother’s socio economic context and experience of pregnancy/ies and postnatal period (not focusing specifically on mental health problems- that is covered in the next section)*

*Please could you share with me some background about your daily life, for example:*

*Do you work?*

*Who are you living with?*

*How many children do you have and how old are they?*

*As mentioned earlier, the reason we are doing this interview with you is to understand your own experiences of mental health care.*

***Map out previous pregnancies and associated mental health problems***

*e.g. tell us a bit about your experience of mental health problems in pregnancy and after the baby was born*

- *Ask participant about:*
  - *Their experience of mental health problems (such as feeling low, worried, difficulties bonding with a baby, unusual experiences/thoughts)*
    - *During pregnancy*
    - *After the baby was born*
  - *(If relevant) In this interview, I will mostly be asking you about your experiences of your most recent pregnancy/childbirth (1-2 years), but please feel free to share with me your previous experiences.*

Potential probes:

- When did these problems start?
- What is your own understanding of this and is there anything that you think may have caused it?
- Tell us about how you have coped?

1. **Experience of perinatal mental health services**

🡪 ***Ask participant about their experience of receiving care from perinatal mental health service (explain which service we are referring to- name of clinicians, explanation i.e. community or MBU etc.) Make sure you cover the experience from initial referral to treatment if help was received. Ask for specific examples wherever possible.***

*By the end of this section, the interviewer should have established how a mum was referred to PMH services and how she accessed services, mum’s experience of that process and care she received and impact of ethnicity/culture on referral process, access and care she received*

**Initial referral including pathway to PMHS**

- When did you or someone else first notice that you needed support/help?
  - Can you let me know a little bit about that please?
- How were you referred (to the first care giver)? What happened next? (Note: interviewer to explore referral pathway)
- Now I would like to explore your experiences of being referred to PMHS
  - What were your initial thoughts about being referred?
  - Did you know about PMHS?
  - What kind of support did you expect to receive from the service?

Did you have any worries or concerns about a referral?

**Accessibility of PMHS (practical elements)**

- I’m going to ask you about what was easy and difficult about accessing PMHS? So first of all, in your experience did you feel it was easy or difficult to attend your appointment?
- Ok, could you let me know what kind of things made it easy/difficult? Let’s start with easy…ok now what was difficult…SPONTANEOUS, THEN PROMPT
  - travel / location
  - time of the day
  - arranging child care
  - language
  - Information about the appointment

**Experiences of using PMHS services**

- Now I would like to talk about the care you received from PMHS. Do you feel like it was what you needed? Can you let me know a little bit more about that.
- What was helpful / less helpful about the care?
- What was good / not so good about the care?
- What did you think of the clinicians that you met with?
- What was the impact of the care you received? SPONTANEOUS, THEN PROMPT:
  - Parenting
  - Mental health
  - Relationships with others
- In your experience was the care/support offered what you needed?

**Acceptability (culture/ethnicity)**

- Research suggests that culture and ethnicity can impact health seeking behaviour – what’s your view on this?
- In your opinion, has your culture/ethnicity been taken into consideration in a referral process and treatment? Can you give me examples of this?
- Do you think that your ethnic background made a difference or influenced your experience of PMHS? SPONTANEOUS, THEN PROBE:
  - Process of seeking help
  - Having a referral made
  - Accessing the service
  - What happened at the service
  - The outcomes from the care received

How do you think your culture/ethnic background is understood by PMHS.

- Do you think PMHS meet the needs of a South Asian/Black woman like yourself?
- Considering your ethnic background, was there anything important missing from your care?
- Have you sought help from somewhere else other than the service, if so, what was your experience?

**Additional Items**

- How would feel about recommending this service to others?
- How do you think the care offered by PMHS could be improved?

**End of interview**

- Any final thoughts or ideas – anything they would like to add
- Thank participants for their time
- Remind re: Confidentiality and recording
- Reimbursements

## Supplementary Material 3. Reflexivity Table

Details of researchers involved in interviewing and analysis.

| **Initials** | **Gender** | **Involvement in study** | **Training** | **Credentials/Education** | **Occupation** | **Other relevant experience/views?** |
| --- | --- | --- | --- | --- | --- | --- |
| MC | Female | Theme-editing; draft write-up | Qualitative analysis trained, Interview training (Social Research Authority). Thematic analysis and Framework analysis training. | BA, MA, MSc, PhD social psychiatry. | Post-doctoral researcher | White Irish ethnicity, migrated age 18 to the UK. Interested in social belonging and its impact on health, migrants’ mental health and critical approaches in psychiatric treatment. |
| SB | Female | Interviewing, analysis of transcripts and creating of matrix, theme editing | Post graduate training in Qualitative research including interviewing, and thematic and framework analysis. | BSc (Hons) Psychology  PGCert Applied Health Research | Research Fellow in Perinatal Mental Health | White British, born and living in the UK with no children. Background in clinical psychology and have worked in various clinical roles in adult mental health in NHS and third sector organisations, including diverse populations in Birmingham and Coventry areas of UK. |
| KCP | Female | Interviewing, analysis of transcripts and creating of matrix | Interviewing, analysis of transcripts and creating of matrix | BSc, MSC, DClinPsy in progress. Training from BSc and MSc in qualitative approaches | Research assistant during data collection, currently trainee clinical psychologist | White British ethnicity, born in Germany and moved to UK before 1st birthday. Interested in developmental psychology and passionate about working with children and young people and their families. |
| HKS | Female | Topic guide development, interviewing, analysis and interpretation and mapping | Qualitative analysis interview training | BA, MA, PHD, AFHEA | Associate Lecturer | Academic, South Asian Britain, Indian, second generation. Interests in health inequities among ethnic minority groups in the perinatal period, lived experiences of illness and patient experiences of healthcare services. PhD in the lived experiences of South Asian women with a severe postnatal psychiatric illness and their experiences of perinatal mental health services. Previous experience of a clinical placement at a Mother and Baby Unit. |
| AS | Female | Interviewing, analysis and interpretation and mapping  co-ordinating LEAP involvement | Framework analysis training | BA Social Work | Social Worker and  Peer researcher | White British with lived experience of postpartum psychosis. |
| KB | Female | Theme editing | Work as a clinical studies officer.  Training from BSc and MRes in qualitative research methods.  Training and delivery of focus groups, delivering interventions and data collection from one-to-one interviews with children. Training and experience of structured and semi-structured interviews. | BSc, MRes in progress | Clinical Research Practitioner | Researcher born in the UK but from a South Asian background. Previous work experience was in Sri Lanka in a mother and baby unit which sparked interest of perinatal services in the UK and wanting to learn more about the services offered in the UK. Worked as an honorary assistant psychologist in perinatal mental health services which increased awareness of the running of the service locally. |

## Supplementary Material 4. Table of Themes and subthemes

| Theme 1. Self-identity, social expectations different attributions of distress, and fear of consequences deter help-seeking | 1.1 Seeking help affects how women see themselves: “It is hard to get your head around being a person that needs help” |
| --- | --- |
|  | 1.2 Minimising distress and self-dismissal: *“It makes you feel like you are complaining too much”* |
|  | 1.3 Different attributions of mental distress affect where women seek help: *“Turning to religion rather than science”* |
|  | 1.4 Shame, discretion, suspicion and fear: *“I was scared that someone’s gonna take my baby away”* |
|  | 1.5 1.5 Others’ judgement and discrimination leave women isolated: “Not good enough” |
| Theme 2. Hidden and disorganised services impede getting support | 2.1 Not knowing what support is available: *“I was like, peri what?”* |
|  | 2.2 Opaque and inconsistent: “*I kind of felt I fell through the cracks a little bit”* |
| Theme 3. The role of curiosity, kindness and flexibility in making women feel heard, accepted and supported by clinicians | 3.1 Services are nurturing: “*I am a different woman, they gave me my self-respect”* |
|  | 3.2 Respected and treated equally, mostly: *“they just saw a mother than needs help”* |
|  | 3.3 Flexibility and curiosity in staff inspire feeling accepted and respected: “*Yes, she understood me, because she asked questions”* |
| Theme 4. A shared cultural background may support or hinder trust and rapport | 4.1 Sharing ethnic background with a clinician is unimportant, and even unhelpful: *“They’re trained in mental health, but they’re not trained in black culture”* |
|  | 4.2 Clinicians and patients sharing a cultural background is a shortcut to rapport: *“It doesn’t make a difference”* |
